# De-escalating Therapy in Obstructive Sleep Apnoea After Glucagon-Like Peptide-1 Receptor Agonist-Induced Weight Loss: A Narrative Review

**DOI:** 10.7759/cureus.116412

**Published:** 2026-09-17

**Authors:** Wares Jalil

**Affiliations:** 1 Respiratory Medicine, University Hospitals of Leicester NHS Trust, Leicester, GBR

**Keywords:** continuous positive airway pressure, deprescribing, glucagon-like peptide-1 receptor agonists, obstructive sleep apnoea, tirzepatide, weight loss

## Abstract

Incretin-based therapies now achieve weight reduction of a magnitude previously attainable only through bariatric surgery and have demonstrated substantial reductions in the apnoea-hypopnoea index (AHI) in adults with obesity-related obstructive sleep apnoea (OSA). Sleep services are consequently managing growing numbers of patients who undergo rapid, substantial weight loss while established on positive airway pressure (PAP) therapy, and who ask whether that therapy can be stopped. No guideline addresses this question.

This narrative review synthesises three literatures that have developed largely in isolation: the relationship between weight change and OSA severity, the efficacy data for glucagon-like peptide-1 receptor agonists and dual incretin agonists in OSA, and the experimental PAP withdrawal literature. Across randomised trials and meta-analyses, there is consistent agreement that incretin therapy reduces AHI, body weight and blood pressure, with effect magnitude varying by agent, dose and baseline adiposity. There is, however, material disagreement on three questions directly relevant to de-escalation: whether the effect is wholly weight-mediated, whether AHI reduction translates into reduction in cardiovascular risk, and what proportion of patients achieve genuine disease remission as opposed to severity reduction. Approximately half of participants in the largest trial did not reach a severity threshold at which PAP would ordinarily be considered unnecessary, and withdrawal studies demonstrate recurrence of obstructive events and adverse haemodynamic change within days of stopping therapy.

This review proposes a structured, reversible and documented framework for reassessment rather than ad hoc discontinuation and identifies the evidence gaps that must be closed before de-escalation can be considered evidence-based practice.

## Introduction and background

Obstructive sleep apnoea (OSA) is among the most prevalent chronic respiratory conditions, and excess adiposity is its principal modifiable aetiological determinant [1,2]. Positive airway pressure (PAP), most commonly delivered as continuous positive airway pressure (CPAP), has been the first-line intervention for moderate-to-severe disease for four decades [1,3], and until recently the alternative of durable, substantial weight loss was realistically available only through bariatric surgery [4,5].

That position has changed. Glucagon-like peptide-1 (GLP-1) receptor agonists and the dual glucose-dependent insulinotropic polypeptide (GIP) and GLP-1 receptor agonist tirzepatide now produce weight reduction of a magnitude that materially alters upper airway anatomy and sleep-disordered breathing severity [6]. Regulatory approval of tirzepatide for moderate-to-severe OSA with obesity, and commissioning arrangements in several health systems that use sleep-disordered breathing as a qualifying comorbidity for funded obesity pharmacotherapy [7], together mean that respiratory and sleep services are now seeing substantial numbers of patients on both therapies simultaneously.

This generates a clinical question that is being asked in practice before it has been addressed in the literature, namely whether PAP can be reduced or withdrawn in a patient who has lost twenty per cent of body weight on incretin therapy, and on what evidential basis such a decision should rest. Existing guidance, including the National Institute for Health and Care Excellence (NICE) guideline on obstructive sleep apnoea/hypopnoea syndrome and obesity hypoventilation syndrome (OHS) in over-16s, its associated intervention evidence review and the corresponding Clinical Knowledge Summary, addresses the initiation of therapy and the demonstration of efficacy at initiation [3,8,9]. It does not anticipate pharmacologically induced weight loss of this magnitude, and it does not describe a withdrawal pathway.

The existing review literature on incretin therapy in OSA is substantial and growing, encompassing narrative reviews [10-12], broader syntheses of the therapeutic role of these agents [13,14], appraisals of their wider effects on sleep disorders [15], a scoping review [16] and several meta-analyses [17-19]. That literature has, however, been concerned almost exclusively with whether these agents work. The distinct and downstream question of what should then happen to establish PAP therapy has not been systematically addressed. This review examines that question.

Scope 

The discussion is restricted to adults with eucapnic, obesity-related OSA. Patients with OHS are explicitly excluded from the framework proposed here, for the reasons set out later in this review; the evidence base in that population is separate, considerably thinner, and the consequences of premature withdrawal are more severe [20].

Methods

This is a narrative review and was not prospectively registered. A structured literature search was performed in PubMed between 2 and 3 August 2026. The primary search string was ("Tirzepatide" OR "glp-1 receptor agonist" OR "glp-1 receptor agonists" OR "incretin") AND ("OSA" OR "obstructive sleep apnoea" OR "obstructive sleep apnea") AND ("CPAP" OR "positive airway pressure"), which returned 50 records. Titles and abstracts were screened against relevance to incretin pharmacotherapy, sleep-disordered breathing severity and PAP use, and 23 articles were selected; these are summarised in Table 1. Because the primary search did not capture the experimental PAP withdrawal literature, diagnostic testing methodology, perioperative guidance, national driving regulation, or background material on the pathophysiology of obesity-related sleep-disordered breathing, the search was supplemented by targeted searching in these areas and by hand-searching of National Institute for Health and Care Excellence guidance [3,8,9], the British Thoracic Society position statement on driving and OSA [21], the European Respiratory Society statement on sleep apnoea, sleepiness and driving risk [22], and the reference lists of identified reviews. Only English-language publications were considered. Consistent with the narrative design, formal risk-of-bias assessment and quantitative synthesis were not undertaken, and the conclusions should be interpreted as an interpretive synthesis rather than a systematic appraisal.

**Table 1:** Summary of the 23 studies identified by the primary literature search. Studies are listed in ascending order of reference number [2,4-7,10-20,23-29] and comprise the full yield of the primary PubMed search described in the Methods. Sources identified subsequently by targeted and hand-searching, comprising national guidance, the positive airway pressure withdrawal literature, diagnostic and perioperative sources and background pathophysiology, are cited in the text but are not tabulated here. AHI, apnoea-hypopnoea index; CPAP, continuous positive airway pressure; GLP-1, glucagon-like peptide-1; OHS, obesity hypoventilation syndrome; OSA, obstructive sleep apnoea; PAP, positive airway pressure.

Study	Design	Population	Intervention or focus	Findings relevant to de-escalation
Soliman et al. [2]	Narrative review	Adults with obesity and respiratory disease	Mechanisms and phenotypes of obesity-related respiratory disease	Describes anatomical and non-anatomical contributors to obesity-related sleep-disordered breathing.
Griffin et al. [4]	Narrative review	Adults with sleep-disordered breathing	Weight management strategies	Weight reduction lowers OSA severity across modalities; the relationship is continuous rather than threshold-based.
Song and Hu [5]	Integrated review	Adults with OSA	Surgical, pharmacological and lifestyle weight loss	Anatomical improvement after surgery is largely maintained without ongoing intervention; pharmacological improvement is not.
Malhotra et al. [6]	Two parallel 52-week double-blind randomised controlled trials (SURMOUNT-OSA)	469 adults, moderate-to-severe OSA and obesity; trial 1 not using PAP (n = 234), trial 2 established on PAP (n = 235)	Tirzepatide 10 or 15 mg weekly versus placebo	AHI treatment difference −20.0 and −23.8 events per hour; 42.2% and 50.2% reached a composite threshold at which PAP might not be recommended; trial 2 embedded a seven-day PAP washout before each polysomnogram.
Albor et al. [7]	Narrative review and prioritisation framework	Adults with obesity-related conditions	GLP-1 receptor agonists and multi-agonist incretin therapies	Sets out prioritisation rationale; notes discontinuation for non-clinical reasons including cost and loss of funded access.
El-Solh et al. [10]	Narrative review	Adults with obesity-related OSA	GLP-1 receptor agonists	Summarises efficacy in obesity-related OSA; does not address withdrawal of established therapy.
Alluri et al. [11]	Comprehensive review	Adults with OSA	GLP-1 receptor agonists	Reviews therapeutic potential and candidate mechanisms of action.
Zheng and Yee [12]	Narrative review	Adults with OSA	GLP-1-based therapy	Contemporary appraisal of the evidence base and its limitations.
Romariz et al. [13]	Review	Adults with OSA and obesity	GLP-1 receptor agonists	Summarises available trial evidence for incretin therapy in OSA.
Jaganathan et al. [14]	Narrative review	Adults with OSA	Pharmacotherapy for OSA	Describes non-anatomical contributors and possible direct effects on ventilatory control and upper airway musculature.
Mifsud et al. [15]	Narrative review	Adults with sleep disorders	GLP-1 receptor agonists	Raises the possibility of effects on sleep-disordered breathing beyond those attributable to weight reduction.
Le et al. [16]	Scoping review	Adults with OSA	GLP-1 receptor agonists	Maps the available literature and identifies evidence gaps.
Li et al. [17]	Systematic review and meta-analysis	Six studies, 1,067 participants	GLP-1 receptor agonists versus comparator	Pooled AHI difference −9.48 events per hour; tirzepatide −21.86 versus liraglutide −5.10; confidence interval crossed the null in the CPAP-treated subgroup; effect not modified by the treatment duration.
Kow et al. [18]	Systematic review and meta-analysis of placebo-controlled randomised trials	Adults with OSA without diabetes	GLP-1 receptor agonists	Confirms direction and approximate magnitude of effect in non-diabetic populations.
Dandamudi et al. [19]	Systematic review and meta-analysis	Adults with OSA	GLP-1 receptor agonists	Cardiometabolic and respiratory outcomes concordant in direction with other syntheses.
Masa et al. [20]	Narrative review	Adults with OHS	Pathophysiology and management of OHS	OHS is a disorder of ventilatory control and respiratory mechanics carrying higher morbidity and mortality than eucapnic OSA.
Blackman et al. [23]	32-week double-blind randomised controlled trial (SCALE Sleep Apnea)	Adults with obesity and moderate or severe OSA	Liraglutide 3.0 mg versus placebo	Significant reduction in AHI alongside weight loss over 30 weeks of treatment.
Wu et al. [24]	Retrospective cohort analysis	Adults with obesity	Semaglutide	Reports effects on OSA risk and downstream outcomes in a real-world population.
Sprung et al. [25]	Randomised controlled trial protocol (ROMANCE)	Adults with type 2 diabetes and OSA	Liraglutide with or without CPAP over 26 weeks	Closest existing design to a direct comparison of pharmacological and mechanical therapy; offers a partial template for a de-escalation trial.
Funamizu and Hirose [26]	Case report	One adult with type 2 diabetes	Tirzepatide	Describes improvement in OSA following tirzepatide-induced weight loss.
Kanu et al. [27]	Secondary analysis of a randomised controlled trial	SURMOUNT-OSA participants	Tirzepatide versus placebo	Improvement in patient-reported sleep-related impairment and sleep disturbance.
Shinde et al. [28]	Qualitative exit interview study	SURMOUNT-OSA participants	Tirzepatide	Patient-reported experience concordant with the quantitative outcomes of the parent trial.
Borissov et al. [29]	Observational cohort study	Adults with OSA and type 2 diabetes	GLP-1 receptor agonists	Observational signal for cardiovascular and pulmonary complications; design cannot establish causation.

## Review

Weight change and obstructive sleep apnoea severity: the pre-incretin evidence base

The dose-dependent relationship between adiposity and OSA severity is long established and is not in dispute. Systematic meta-analysis of pooled weight-loss interventions indicates that an initial ten per cent reduction in body mass index (BMI) is associated with a greater than twenty per cent reduction in the apnoea-hypopnoea index (AHI) and that this relationship is broadly consistent irrespective of the modality by which weight loss is achieved [30], an observation reiterated in subsequent meta-analytic work in this field [17]. Reviews of the role of weight management in sleep-disordered breathing, and integrated comparisons of surgical, pharmacological and lifestyle strategies, converge on the same conclusion: weight loss reduces OSA severity, and the relationship is continuous rather than threshold-based [4,5].

Two features of this literature are frequently underweighted when it is invoked to justify stopping therapy.

The first is the distinction between severity reduction and disease remission. A patient whose AHI falls from 50 to 20 events per hour has experienced a substantial and clinically meaningful improvement and remains in the moderate severity category for which PAP is conventionally indicated [1,3]. Mean effect sizes describe the former; de-escalation decisions require the latter.

The second is heterogeneity of response. Obesity influences upper airway collapsibility through parapharyngeal and lingual fat deposition, reduced lung volumes and altered chest wall mechanics [2,4], but OSA is not a purely anatomical disorder. Reviews of the pathophysiology of obesity-related respiratory disease [2], of the pathophysiology of adult OSA [31] and of the emerging pharmacotherapy of the condition [14] describe non-anatomical contributors, including ventilatory control instability, low arousal threshold and impaired upper airway dilator muscle responsiveness. The extent to which pharmacologically induced weight loss modifies each of these components, and how uniformly it does so across individuals, is the subject of the evidence considered next.

Efficacy of incretin therapies in obstructive sleep apnoea: where the evidence agrees

The randomised evidence is dominated by two agents.

The SCALE Sleep Apnea trial randomised individuals with obesity and moderate or severe OSA to liraglutide 3.0 mg or placebo over 30 weeks and demonstrated significant reduction in AHI alongside weight loss [23]. The SURMOUNT-OSA programme subsequently randomised 469 adults with moderate-to-severe OSA and obesity across two parallel 52-week phase 3 trials: trial 1 enrolled participants unable or unwilling to use PAP (n = 234), and trial 2 enrolled participants established on PAP for at least three months who planned to continue it (n = 235) [6]. Mean baseline AHI was 51.5 and 49.5 events per hour and mean BMI 39.1 and 38.7 respectively. At 52 weeks, the estimated treatment difference in AHI was −20.0 events per hour (95% confidence interval [CI] −25.8 to −14.2) in trial 1 and −23.8 events per hour (95% CI −29.6 to −17.9) in trial 2, corresponding to percentage reductions of 50.7% and 58.7% from baseline. Body weight fell by 17.7% and 19.6% [6].

Beyond these two programmes, evidence for semaglutide in this population derives from analysis of OSA risk and outcomes in patients with obesity [24], a randomised trial of liraglutide with and without concurrent CPAP in type 2 diabetes and OSA has been protocolised and provides the closest existing design to a direct comparison of pharmacological and mechanical therapy [25], and case-level reports describe improvement in OSA following tirzepatide-induced weight loss [26].

Meta-analytic synthesis is consistent in direction while differing in magnitude according to the agents pooled. The meta-analysis by Li and colleagues, incorporating six studies and 1,067 participants, estimated a pooled AHI treatment difference of −9.48 events per hour (95% CI −12.56 to −6.40), with weight reduction of −10.99 kg, systolic blood pressure reduction of −4.81 mmHg and diastolic reduction of −0.32 mmHg [17]. Subgroup analysis found tirzepatide substantially more effective than liraglutide (−21.86 versus −5.10 events per hour), and participants with obesity deriving greater benefit than those without (−12.93 versus −4.31 events per hour) [17]. Further meta-analyses restricted to non-diabetic populations and to placebo-controlled randomised trials [18], and additional syntheses of cardiometabolic and respiratory outcomes [19], report findings in the same direction.

There is therefore no serious disagreement in the literature that incretin therapy reduces AHI, body weight and blood pressure in obesity-related OSA, nor that the magnitude of AHI reduction tracks the potency of the agent and the degree of baseline adiposity [17-19]. Beyond this point, the agreement ends.

Where the evidence diverges: three unresolved issues with direct bearing on withdrawal

The Extent to Which the Effect is Weight-Mediated

The dominant explanatory model attributes the reduction in AHI to reduced parapharyngeal and central adiposity secondary to appetite suppression [4,5]. Several observations sit uneasily with an exclusively weight-mediated account. Li and colleagues note that liraglutide reduced OSA severity in cohorts with mean BMI in the overweight rather than obese range and that in one short four-week study no significant correlation was observed between AHI improvement and change in BMI [17]. They further note preclinical evidence that GLP-1 receptor activation may influence respiratory drive and breathing stability [17], and reviews of the emerging pharmacotherapy of OSA and of GLP-1 effects on sleep disorders raise the same possibility of direct effects on ventilatory control and upper airway musculature [14,15].

The subgroup analysis in the same meta-analysis found that treatment duration between four and 52 weeks did not significantly modify effect size, implying that benefit accrues early [17]. This is often presented as reassuring. For the withdrawal question, it is ambiguous: an effect that appears within four weeks and is not fully explained by weight change may also dissipate rapidly on discontinuation, even if weight is maintained. This possibility has not been tested.

The clinical implication is direct. If part of the benefit is pharmacological rather than anatomical, then a patient who stops the drug while maintaining weight may nonetheless experience deterioration in sleep-disordered breathing. Any de-escalation framework must therefore treat drug discontinuation, not merely weight regain, as a trigger for reassessment.

The Relationship Between Apnoea-Hypopnoea Index Reduction and Cardiovascular Risk

This is the most consequential discrepancy in the field and it is insufficiently discussed in the existing reviews.

SURMOUNT-OSA reported improvement across a range of risk-relevant secondary endpoints: sleep apnoea-specific hypoxic burden fell by −70.1 and −61.3 % min/hr relative to placebo, high-sensitivity C-reactive protein fell, and systolic blood pressure fell by 7.6 and 3.7 mmHg [6]. Hypoxic burden is argued to capture cardiovascular risk more faithfully than AHI, on the grounds that a frequency-based metric does not reflect the depth and duration of respiratory event-related desaturation; in analyses of the Osteoporotic Fractures in Men Study and the Sleep Heart Health Study, hypoxic burden predicted cardiovascular disease-related mortality [32]. Analyses of patient-reported outcomes from the same trial programme [27], and qualitative exit interviews with participants [28], describe corresponding improvement in sleep-related impairment and sleep disturbance.

Against this, a randomised proof-of-concept study found that CPAP, but not GLP-1-mediated weight loss, improved markers of early cardiovascular disease in OSA [33]. Studies of cardiovascular and pulmonary complications in patients with OSA and type 2 diabetes receiving GLP-1 receptor agonists provide observational signal but cannot resolve the question [29]. The two findings are not necessarily irreconcilable, since they measure different endpoints over different intervals, but they cannot both be used uncritically to support the proposition that pharmacological AHI reduction is an adequate substitute for mechanical therapy.

It should also be noted that the meta-analytic subgroup comparing participants receiving concurrent CPAP with those not receiving it produced a confidence interval crossing the null in the CPAP-treated group (−10.16, 95% CI −24.52 to 4.19) [17]. The conclusion that the therapeutic effect is unaffected by concurrent CPAP is therefore a statement about the absence of a demonstrated interaction rather than a demonstration of equivalence, and it is a weaker foundation for de-escalation than it is sometimes taken to be.

The proportion of patients achieving remission

Here the trial data can be read more precisely than they usually are, and the reading is central to this review.

SURMOUNT-OSA prespecified, as a multiplicity-controlled secondary endpoint, the proportion of participants achieving an AHI below 5 events per hour, or an AHI of 5 to 14 events per hour with an Epworth Sleepiness Scale (ESS) score of 10 or less [6]. The trial investigators explicitly characterised this composite as representing a severity level at which PAP therapy might not be recommended [6]. In other words, this endpoint is the closest existing approximation to a remission criterion, and it was designed with the treatment-withdrawal question in view.

The proportions reaching it were 42.2% in trial 1 and 50.2% in trial 2, against 15.9% and 14.3% on placebo [6]. The corollary deserves equal emphasis: approximately 58% and 50% of participants receiving a highly effective incretin therapy for 52 weeks, and losing approximately a fifth of their body weight, did not reach a threshold at which withdrawal of PAP could be contemplated. Similarly, while 61.2% and 72.4% achieved at least a 50% reduction in AHI [6], a halving of a baseline AHI of 50 events per hour leaves residual moderate-to-severe disease. The contributors that plausibly account for this residual burden are set out in Table 2.

**Table 2:** Predictors and correlates of residual obstructive sleep apnoea despite substantial weight loss. Synthesised from references [1,2,4,6,14,15,17,30,31,34-36]. Traits are grouped by mechanism rather than by strength of evidence. The final column distinguishes correlates for which direct supporting data are cited in this review from those advanced on mechanistic grounds or extrapolated from populations not undergoing weight loss. No predictor listed has been prospectively validated as a discriminator of durable remission following pharmacological weight loss, and the table should be read as a structured summary of considerations rather than as a risk-stratification instrument. AHI, apnoea-hypopnoea index; CPAP, continuous positive airway pressure; REM, rapid eye movement.

Domain	Trait or correlate	Relevance to de-escalation	Evidence basis in this review
Anatomical contributors			
Anatomical	Craniofacial and skeletal restriction	Retrognathia, maxillomandibular deficiency and a small bony airway are unaltered by weight loss, so collapsibility persists at any weight.	Mechanistic; excluded from pivotal trial population [6,31]
	Residual parapharyngeal and lingual fat	Regional fat loss is not proportional to total body weight loss, so upper airway calibre may improve less than body mass index suggests.	Mechanistic [2,4]
	Baseline disease severity	A 50 per cent reduction from a baseline AHI of approximately 50 events per hour leaves residual moderate-to-severe disease. Higher baseline severity therefore predicts persistent disease despite a large relative treatment effect.	Directly supported [6,30]
	Magnitude of weight loss achieved	An initial 10 per cent reduction in body mass index is associated with a greater than 20 per cent reduction in AHI, so smaller degrees of weight loss predict proportionally smaller improvement.	Directly supported [30]
	Degree of baseline adiposity	Treatment effect on AHI is larger in participants with obesity than in those without, so patients with lower baseline body mass index derive less absolute benefit.	Directly supported [17]
	Nasal obstruction and tonsillar hypertrophy	Fixed upper airway obstruction is not addressed by adiposity reduction.	Mechanistic [1,31]
Non-anatomical (endotypic) contributors			
Non-anatomical	Ventilatory control instability (high loop gain)	Unstable chemoreflex control perpetuates cyclical events independently of airway calibre and is not corrected by weight reduction.	Mechanistic [2,14,31]
	Low respiratory arousal threshold	Premature arousal prevents stable recruitment of upper airway dilator muscles and sustains fragmentation despite improved anatomy.	Mechanistic [31]
	Impaired upper airway dilator muscle responsiveness	Reduced genioglossus recruitment during sleep leaves a smaller anatomical improvement insufficient to maintain patency.	Mechanistic [14,31]
	Possible loss of a weight-independent drug effect	If part of the observed benefit is pharmacological rather than anatomical, sleep-disordered breathing may deteriorate on drug discontinuation even where weight is maintained. The existence and magnitude of such an effect is unresolved.	Hypothesis-generating [14,15,17]
Demographic, methodological and other correlates			
Other	Older age	Identified as a predictor of clinical relapse following CPAP withdrawal. Extrapolation to the post-weight-loss setting is untested.	Indirect; withdrawal cohort [34]
	Larger neck circumference	Identified as a predictor of clinical relapse following CPAP withdrawal. Extrapolation to the post-weight-loss setting is untested.	Indirect; withdrawal cohort [34]
	Positional and REM-related disease	Residual events may concentrate in supine sleep or REM, and may be underestimated by a single diagnostic night.	Mechanistic [1]
	Diagnostic modality and night-to-night variability	Home sleep apnoea testing systematically underestimates AHI relative to attended polysomnography, and a single night may misclassify a patient close to a decision threshold.	Directly supported [1,35]
	Hypopnoea scoring convention	Whether an individual crosses a threshold depends materially on the scoring rule applied. SURMOUNT-OSA used American Academy of Sleep Medicine rule 1B.	Directly supported [6]
	Anticipated discontinuation of incretin therapy	Weight regain follows withdrawal of treatment, so patients unlikely to remain on therapy are poor candidates for de-escalation irrespective of current severity.	Directly supported [36]

Two methodological features of trial 2 make it more informative for the present question than has been recognised. First, participants were established PAP users. Second, and critically, they were instructed to suspend PAP for seven days before each polysomnographic assessment, in order to remove the confounding effect of therapy on the measurement [6]. Trial 2 therefore constitutes, in effect, a structured seven-day supervised therapy holiday followed by objective retesting, performed at 52 weeks in a cohort undergoing substantial pharmacological weight loss. That is precisely the assessment procedure a de-escalation pathway would require, and it is the strongest available empirical anchor for one.

Its limitations must be stated with equal clarity. Trial 2 was not designed to evaluate PAP withdrawal, this was not a prespecified endpoint, participants resumed therapy after assessment, and no outcomes were collected on those who might have stopped [6]. The trial tells us how many patients reached a threshold; it tells us nothing about what happens to those who then act on it.

What the withdrawal literature contributes

The experimental CPAP withdrawal paradigm, developed as a model for studying the physiological consequences of untreated OSA, provides the counterweight to the efficacy data and is largely absent from the existing incretin reviews [37].

Schwarz, Stradling and Kohler’s synthesis of this literature describes a consistent pattern [37]. Withdrawal of effective therapy produces recurrence of obstructive events within a small number of nights, return of subjective sleepiness, increases in morning systolic and diastolic blood pressure and heart rate, increased urinary catecholamine excretion, and measurable deterioration in endothelial function assessed by flow-mediated dilatation, with these changes evident over two weeks. Markers of systemic inflammation, insulin resistance and lipid profile were less consistently affected over that interval [37].

More recent randomised data are concordant. Wali and colleagues randomised patients to complete or intermittent CPAP discontinuation and observed rapid recurrence of OSA within one week in both groups, with significant increases in AHI, oxygen desaturation index and time spent with oxygen saturation below 90% [34]. Clinical relapse occurred at a median of 2.9 days in the intermittent-use group and 3.5 days in the complete-withdrawal group, with age and neck circumference identified as predictors of relapse [34]. The sample was small and the population was not undergoing weight loss, and the findings therefore cannot be transposed directly. They nonetheless establish two points of principle relevant to any withdrawal decision: relapse, where it occurs, is rapid rather than gradual, and intermittent use should not be assumed to be a safe intermediate state.

Synthesising the efficacy and withdrawal evidence considered earlier in this review, the position is as follows. Incretin therapy reliably reduces OSA severity [17-19]; roughly half of treated patients reach a severity threshold at which PAP might not be indicated [6]; and in patients who have not reached such a threshold, cessation of therapy can be expected to produce recurrence of disease and adverse haemodynamic change within days [34,37]. The clinical task is therefore to distinguish the two groups reliably, and to do so before rather than after therapy is stopped.

Weight regain, drug discontinuation and the reversibility problem

Any framework must account for the fact that pharmacological weight loss is contingent on continued treatment. The SURMOUNT-4 randomised withdrawal trial demonstrated that, following an open-label lead-in period of tirzepatide treatment, participants randomised to placebo regained a substantial proportion of the weight lost, whereas those continuing treatment achieved further reduction [36]. Discontinuation occurs commonly for reasons that are not clinical, including cost, supply, tolerability and loss of funded access [7]. Reviews of the prioritisation of incretin therapies for specific obesity-related conditions [7], and of the comparative durability of surgical versus pharmacological weight loss [5], both emphasise this asymmetry: the anatomical improvement conferred by surgery is largely maintained without ongoing intervention, whereas that conferred by pharmacotherapy is not.

The practical consequence is that PAP withdrawal following incretin-induced weight loss should be conceptualised as conditional and reversible rather than as discharge. This has three operational implications: equipment should be retained rather than returned for a defined period; the patient should leave the consultation with explicit criteria for restarting; and cessation of the weight-loss therapy should itself trigger clinical review rather than being managed independently by the prescriber.

A proposed framework for structured de-escalation

The framework summarised in Figure 1 is offered as a structure for making a decision that clinicians are already required to make, not as a guideline. No threshold below is derived from randomised evidence of de-escalation, because none exists. Each is a consensus-free judgement, stated explicitly here rather than left to imply an evidence base that does not exist.

**Figure 1:**
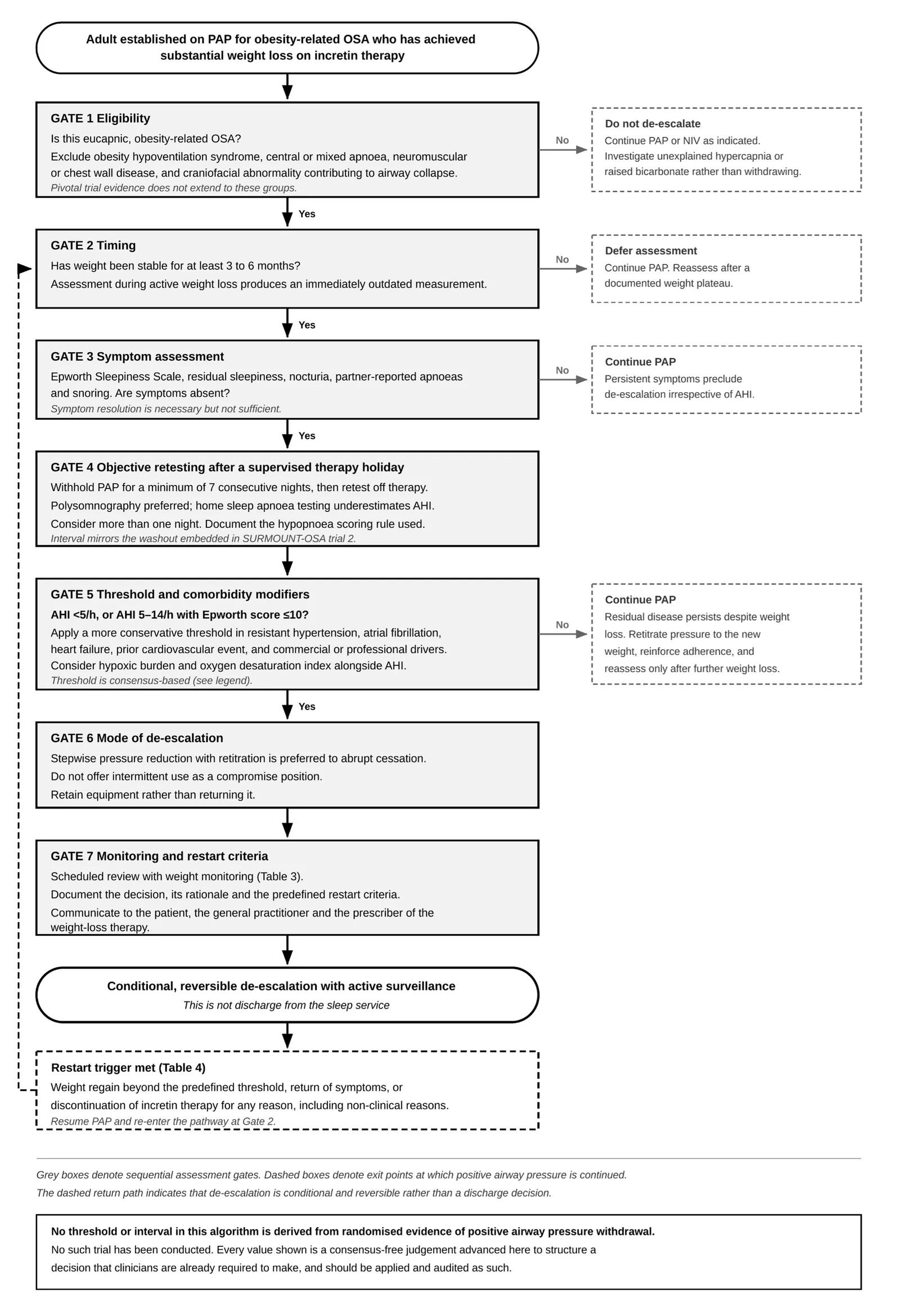
A proposed seven-gate framework for de-escalating positive airway pressure following incretin-induced weight loss. Synthesised from references [1,3,6,7,20,22,32,34-37]. Sequential assessment gates (grey) are applied in order to an adult established on positive airway pressure (PAP) for obesity-related obstructive sleep apnoea (OSA) who has achieved substantial weight loss on a glucagon-like peptide-1 receptor agonist or dual incretin agonist. Failure at any gate directs the patient to an exit point (dashed) at which PAP is continued. Gate 1 excludes obesity hypoventilation syndrome and other phenotypes to which the pivotal trial evidence does not extend. Gate 2 requires a documented weight plateau, since assessment during active weight loss is immediately outdated. Gate 3 establishes symptom resolution, which is necessary but not sufficient. Gate 4 requires objective retesting off therapy after a supervised holiday of at least seven nights, mirroring the washout embedded in the SURMOUNT-OSA trial 2 protocol. Gate 5 applies a severity threshold derived from the composite secondary endpoint of that trial, lowered where cardiovascular comorbidity or a safety-critical occupation is present. Gate 6 favours stepwise pressure reduction over abrupt cessation and excludes intermittent use. Gate 7 establishes scheduled surveillance and predefined criteria for resuming therapy. The dashed return path denotes that de-escalation is conditional and reversible: discontinuation of incretin therapy, weight regain or symptom recurrence returns the patient to Gate 2. No threshold or interval shown is derived from randomised evidence of PAP withdrawal following pharmacological weight loss, because no such trial has been conducted. Each is a consensus-free judgement advanced to structure a decision clinicians already face, and the framework should not be read as a clinical guideline or validated pathway. AHI, apnoea-hypopnoea index; NIV, non-invasive ventilation; OSA, obstructive sleep apnoea; PAP, positive airway pressure.

Gate 1: Eligibility

This is original diagnosis of obesity-related, eucapnic OSA. Patients with OHS, significant central or mixed apnoea, neuromuscular or chest wall disease, or craniofacial abnormality contributing to airway collapse were excluded [3,20]. Note that the pivotal trial excluded central and mixed sleep apnoea and major craniofacial abnormality, so its findings do not extend to these groups [6].

Gate 2: Timing

Assessment should follow a period of weight stability rather than being undertaken during active weight loss, when any measurement is immediately outdated. A plateau of at least three to six months is proposed. The pivotal trial required weight stability of within 5 kg over the three months preceding enrolment [6], which offers some precedent for this order of magnitude.

Gate 3: Symptom Assessment

Assessment comprises the ESS, residual sleepiness, nocturia, and partner-reported apnoeas and snoring [1]. Symptom resolution is necessary but not sufficient, since asymptomatic patients with moderate-to-severe disease retain cardiovascular risk [6,32].

Gate 4: Objective Retesting After a Supervised Therapy Holiday

Retesting must be performed off therapy. A minimum seven-day period off PAP before assessment is proposed, consistent with the washout used in SURMOUNT-OSA trial 2 [6] and with the observation from withdrawal studies that relapse manifests within this window [34,37]. Attended polysomnography remains the reference standard. Home sleep apnoea testing is an accepted alternative for diagnosis in appropriately selected patients, but its recognised limitations bear directly on a de-escalation decision: because sleep is not recorded, the AHI is derived from total recording time rather than total sleep time and is systematically underestimated, and misclassification is most consequential at precisely the low severities on which the decision turns [1,35]. Where home testing is used for capacity reasons, the risk of underestimation should be explicitly weighed, a negative or borderline study in a symptomatic patient should prompt attended polysomnography, and consideration should be given to testing on more than one night in view of night-to-night variability [35]. Hypopnoea scoring criteria should be documented, since these materially affect derived AHI; SURMOUNT-OSA used the American Academy of Sleep Medicine rule 1B, requiring a 30% or greater reduction in airflow for at least 10 seconds with 4% or greater desaturation [6].

Gate 5: Thresholds and Comorbidity Modifiers

The SURMOUNT-OSA composite is proposed as the reference threshold, namely AHI below 5 events per hour, or AHI of 5 to 14 with an ESS score of 10 or less, on the grounds that it is the only threshold in the literature explicitly constructed to denote a level at which PAP may not be required [6]. Greater caution is warranted, and a lower threshold should be applied, in resistant hypertension, atrial fibrillation, heart failure, prior cardiovascular events, and in commercial or professional drivers [1,3,22]. Consideration should be given to hypoxic burden and oxygen desaturation index alongside AHI, given the evidence that these better reflect cardiovascular risk [6,32].

Gate 6: Mode of De-escalation

Where feasible, stepwise pressure reduction with retitration is preferable to abrupt cessation. Intermittent use should not be offered as a compromise, since the available randomised data suggest that it does not confer protection against relapse [34].

Gate 7: Monitoring and Restart Criteria

Review at defined intervals with weight monitoring was done following the schedule proposed in Table 3. Predefined restart triggers, together with the threshold and required response for each, are set out in Table 4. They should include weight regain beyond a specified threshold, return of symptoms, and discontinuation of incretin therapy for any reason [7,36]. Equipment should be retained for a defined period. The decision, its rationale and the restart criteria should be documented and communicated to the patient, the general practitioner and the prescriber of the weight-loss therapy.

**Table 3:** Proposed surveillance schedule following de-escalation of positive airway pressure. Synthesised from references [6,7,21,22,32,34-38]. The schedule sets out the minimum review points proposed following a decision to reduce or withdraw positive airway pressure at the final gate of the framework proposed in this review. The week 0 entry establishes the reference dataset against which all subsequent change is judged, and should be recorded before therapy is reduced. The week 4 review is placed early because the withdrawal literature indicates that symptomatic relapse, where it occurs, manifests within days rather than months. Confirmation at each visit that incretin therapy is continuing at an effective dose is included because pharmacological weight loss is contingent on continued treatment, and because prescribing may sit outside the respiratory service. Repeat objective testing is proposed at 12 months irrespective of symptoms, since symptom-based surveillance alone will not detect asymptomatic recurrence carrying cardiovascular risk. Retention of equipment is an operational recommendation intended to permit immediate resumption without a new referral, and is not derived from published evidence. No interval in this table is derived from randomised evidence of positive airway pressure withdrawal following pharmacological weight loss. Each is a consensus-free judgement advanced here to structure surveillance that would otherwise be undertaken ad hoc. Local adaptation to service capacity is expected, and any adopted schedule should be audited prospectively. AHI, apnoea-hypopnoea index; PAP, positive airway pressure.

Timepoint	Assessment	Purpose	Action if abnormal
Week 0 (decision)	Confirm Gates 1 to 5 satisfied. Record weight, body mass index, Epworth score, off-therapy AHI, oxygen desaturation index, hypopnoea scoring rule and current incretin agent and dose. Issue written restart criteria.	Establish the reference dataset against which all later change is judged, and transfer the restart criteria to the patient.	Do not proceed; return to the relevant gate.
Week 4	Telephone or virtual review. Symptom enquiry, Epworth score, weight.	Detect early symptomatic relapse, which withdrawal studies indicate occurs within days rather than months.	Resume PAP and re-enter at Gate 2.
Month 3	Clinical review. Weight, Epworth score, partner-reported symptoms, blood pressure. Confirm incretin therapy continuing at effective dose.	Detect early weight regain and confirm the pharmacological basis of the anatomical improvement is intact.	Resume PAP; consider repeat objective testing.
Month 6	As month 3, plus review of cardiovascular comorbidity and, for drivers, documented confirmation of continued fitness.	Address the comorbidity and occupational obligations that a withdrawal decision does not suspend.	Resume PAP; notify licensing authority if applicable.
Month 12	Clinical review with weight, Epworth score and blood pressure. Consider repeat objective sleep study irrespective of symptoms.	Symptom-based surveillance alone will not detect asymptomatic recurrence carrying cardiovascular risk.	Resume PAP; retitrate to current weight.
Annually thereafter	Weight, symptoms and blood pressure, with objective retesting prompted by any restart trigger.	Maintain the patient within a defined service rather than discharging.	Resume PAP and re-enter at Gate 2.
Equipment	Retain device and mask for a minimum of 12 months rather than returning to stores.	Permits immediate resumption without a new referral or reissue delay.	Not applicable.

**Table 4:** Restart triggers following de-escalation of positive airway pressure. Synthesised from references [6,7,20-22,32,34,36-40]. Triggers are listed in the order of priority proposed here rather than by expected frequency, and any one of them is sufficient to prompt the stated response. Discontinuation of incretin therapy is placed first because weight regain follows withdrawal of treatment, and because any weight-independent component of the therapeutic effect would also be lost; recognising this trigger requires an explicit communication route to the prescriber of the weight-loss therapy. The weight regain threshold is set to correspond to the weight stability margin required before de-escalation is considered and has no independent empirical basis. The symptom threshold reflects the observation that relapse after therapy withdrawal is rapid, so resumption of therapy is advised without waiting for an objective study. The final entry is not a relapse trigger: unexplained hypercapnia indicates a different disease process and should prompt investigation for hypoventilation rather than resumption of therapy for eucapnic disease. No threshold in this table is derived from randomised evidence of positive airway pressure withdrawal following pharmacological weight loss. Each is a consensus-free judgement advanced here. Thresholds should be recorded in the notes and issued to the patient in writing at the time of de-escalation, so that the criteria for resuming therapy are agreed in advance rather than determined retrospectively. ESS, Epworth Sleepiness Scale; OSA, obstructive sleep apnoea; PAP, positive airway pressure.

Trigger	Proposed threshold	Rationale and required response
Discontinuation of incretin therapy	Any cessation or sustained dose interruption, for clinical or non-clinical reasons including cost, supply, tolerability or loss of funded access.	Weight regain follows treatment withdrawal, and any weight-independent component of the effect would also be lost. This is the single most important trigger and must be communicated to the prescriber. Resume PAP and re-enter at Gate 2.
Weight regain	Return of 5 kg, or 5 per cent of the weight lost, above the weight recorded at the time of de-escalation.	Selected to correspond to the stability margin applied at Gate 2. Prompt clinical review and consider objective retesting.
Return of symptoms	Epworth score of 11 or more, or recurrence of witnessed apnoeas, unrefreshing sleep, nocturia or morning headache.	Symptomatic relapse after withdrawal occurs within days. Resume PAP without waiting for an objective study.
New or worsening cardiovascular comorbidity	New atrial fibrillation, worsening blood pressure control, incident heart failure or an acute cardiovascular event.	Lowers the acceptable residual disease threshold applied at Gate 5. Resume PAP and reassess.
Sleepiness-related driving event or near miss	Any such event, self-reported or otherwise.	Resume PAP immediately, advise the patient of licensing obligations and document the advice given.
Planned surgery or procedural sedation	Any procedure requiring sedation or general anaesthesia.	Historical moderate-to-severe OSA should be declared and managed as active until objectively excluded. Incretin therapy should be declared separately because of delayed gastric emptying. Consider perioperative resumption of PAP.
Emergence of hypercapnia	Unexplained daytime hypercapnia or raised serum bicarbonate.	Indicates a different disease process. Do not manage as relapse of eucapnic OSA; investigate for hypoventilation.

Situations in which de-escalation should not be considered

Obesity Hypoventilation Syndrome and Suspected Hypoventilation

OHS is a disorder of ventilatory control and respiratory mechanics rather than of upper airway collapsibility alone, and carries substantially higher morbidity and mortality than eucapnic OSA [20]. It is addressed separately in national guidance [3,8]. Critically, patients with hypercapnic respiratory failure were not represented in the pivotal incretin trials, and no trial has reported daytime arterial carbon dioxide tension, serum bicarbonate or ventilatory response as an outcome [6,20]. Improvement in AHI in this group cannot be assumed to indicate correction of hypoventilation. Unexplained hypercapnia or elevated bicarbonate should prompt investigation, not withdrawal [20].

Drivers and Safety-Critical Occupations

The European Respiratory Society statement on sleep apnoea, sleepiness and driving risk sets out the relationship between untreated disease, sleepiness and collision risk, and the expectations placed on clinicians assessing fitness to drive [22]. In the United Kingdom, British Thoracic Society position guidance on driving and obstructive sleep apnoea [21] and Driver and Vehicle Licensing Agency (DVLA) requirements on excessive sleepiness and driving [38] together govern practice and place obligations on both cliniciasn and patients that are not suspended by pharmacological weight loss. Any de-escalation in this group requires documented objective retesting off therapy and explicit communication of licensing obligations, since an undocumented withdrawal decision carries obvious medicolegal exposure.

Perioperative and Periprocedural Contexts

Two considerations coincide. A patient with historical moderate-to-severe OSA no longer using PAP requires the same anaesthetic precautions as one still receiving it until objectively demonstrated otherwise. Separately, incretin therapy delays gastric emptying, and treated patients may retain considerable gastric contents despite standard preoperative fasting, raising concern about regurgitation and pulmonary aspiration at induction of, or emergence from, anaesthesia [39]. The magnitude of this effect is not uniform across the drug class: short-acting agonists produce the most pronounced delay, whereas long-acting agents exert residual effects that depend on dose and treatment duration [39], and continued treatment appears to attenuate the delay, such that particular caution is advised in patients recently commenced on therapy [40]. The absolute risk of aspiration under general anaesthesia remains unquantified, and recommendations are correspondingly precautionary rather than evidence-based [40]. For the purposes of this review, the point is procedural rather than pharmacological: a patient presenting for sedated endoscopy, dental treatment or surgery may now carry both an unrecognised historical diagnosis of OSA and an active incretin prescription, and both should be declared and managed together.

Service, governance and equity considerations

A structured reassessment pathway carries a resource cost that falls on sleep services already under pressure. Each de-escalation assessment requires a supervised therapy holiday, an objective study, and clinician time to interpret and document [6,37]. Where commissioning arrangements make sleep-disordered breathing a qualifying comorbidity for funded obesity pharmacotherapy, the volume of patients entering this pathway is determined by decisions taken outside the respiratory service, and capacity planning should reflect this [7]. The choice of retesting modality is itself a capacity decision with diagnostic consequences, since the throughput advantage of home testing is obtained at the cost of a systematic underestimate of severity [1,35].

Governance is presently ambiguous. The prescriber of the weight-loss therapy, the sleep physician and primary care may each reasonably assume that another holds responsibility for the PAP decision, with the risk that no reassessment occurs and patients discontinue therapy unilaterally and unmonitored [9]. Explicit local allocation of responsibility is a prerequisite for safe practice.

Finally, access to incretin therapy is unevenly distributed, and OSA outcomes already vary with socioeconomic position. A two-tier situation in which some patients access disease-modifying weight loss while others do not is a foreseeable consequence of current arrangements and warrants monitoring [7].

Evidence gaps

The central gap is the absence of any trial in which PAP has been prospectively and randomly withdrawn following pharmacologically induced weight loss. Every recommendation in this review, including the framework proposed above, is therefore an extrapolation from trials designed to answer a different question [6,23]. Specific priorities follow. First, randomised comparison of structured de-escalation against continued PAP in patients meeting remission thresholds after incretin therapy, powered for cardiovascular and symptom endpoints rather than AHI alone; the protocolised comparison of liraglutide with and without CPAP offers a partial design template [25]. Second, characterisation of the durability of AHI improvement after incretin discontinuation, distinguishing the contribution of weight regain from any loss of weight-independent effect, since the existence and magnitude of the latter remains unresolved [17,36]. Third, validation of predictors of durable remission, including endotypic traits, so that reassessment can be targeted rather than universal [2,31]. Fourth, determination of the appropriate retesting modality, timing and off-therapy interval, given that diagnostic modality, night-to-night variability and hypopnoea scoring conventions each materially affect whether an individual patient crosses a threshold [1,6,35]. Fifth, evidence in OHS, from which this population has been wholly excluded and in which the consequences of premature withdrawal are gravest [20]. Until these questions are addressed, de-escalation of PAP after pharmacological weight loss should be undertaken as a structured, documented and reversible clinical decision, and should be recognised as one taken in the absence of direct supporting evidence.

## Conclusions

Substantial weight loss changes the treatment question in OSA, but it does not answer it. The largest trial of incretin therapy in this population more than halved the AHI, yet approximately half of participants still did not reach a severity at which withdrawal of PAP could reasonably be contemplated. Improvement and remission are not the same endpoint, and only the second supports stopping treatment. Where remission has not occurred, the withdrawal literature indicates that recurrence of obstructive events and adverse haemodynamic change follow within days rather than months. The practical implication is that de-escalation should be treated as a conditional and reversible clinical decision rather than as discharge from the sleep service, undertaken only after objective retesting off therapy, accompanied by predefined restart criteria, and framed so that discontinuation of the weight-loss therapy is itself recognised as a trigger for reassessment. Until the question is addressed directly by trial, the case for a structured pathway rests not on evidence of its safety but on the transparency, documentation and auditability it confers.
